# **Implementing a National PrEP Program:** How Can We Make It Happen?

**DOI:** 10.1017/jme.2022.36

**Published:** 2022

**Authors:** David Malebranche, Ariel Watriss, Derek T. Dangerfield

**Keywords:** HIV, Prevention, Sexual Health, Structural

## Abstract

Inequities in HIV pre-exposure prophylaxis (PrEP) use persist in the United States. Although scientific advancement in delivery options and social acceptance of PrEP has occurred in the past decade, gaps remain in ensuring that this sexual health program is available to all. Components of what a national PrEP program for all would look like are discussed.

Pre-exposure prophylaxis, or “PrEP,” is a sexual health program intended to prevent individuals from acquiring HIV (Centers for Disease Control and Prevention 2014). In 2012, the U.S. Food and Drug Administration (FDA) approved tenofovir disoproxil fumarate (TDF) + emtricitabine (FTC), commonly known by the brand name Truvada, as the first oral regimen for daily PrEP use due to persuasive studies demonstrating effectiveness among sexual minority men (SMM) who have sex with men, transwomen (TW), heterosexual serodiscordant couples, and people who inject drugs (PWID).^1^ Additional studies among SMM and TW showed equal effectiveness and potentially less renal and bone side effects as Truvada and led to the FDA approval of tenofovir alafenamide (TAF) + FTC, known by the brand name Descovy, for PrEP use in 2019.^2^ However, Descovy is not FDA-approved for persons engaging in receptive vaginal sex and has been linked to potential weight gain and elevated cholesterol levels.^3^ In December 2021, the FDA approved a long-acting injectable version of PrEP called cabotegravir, known by the brand name Apretude, which is administered as an intramuscular shot every two months and is equally effective, if not better at preventing HIV acquisition, than both the approved oral regimens.

Despite increasing PrEP use since it was first approved in 2012, these biomedical advancements in HIV prevention have not translated into equitable access to the populations who need it the most.^4^ Specifically, Black and Latino SMM, TW, and Black cisgender women experience suboptimal access, use, and adherence to PrEP given the high need for prevention in these subpopulations.^5^ Access, use, and adherence are particularly low in the U.S. South.^6^ Well-known individual-, social-, clinical-, and structural-level barriers to PrEP use include low awareness and perceived need, anticipated and experienced stigma from clinicians, partners, and peers, clinician bias and low prescriptions, and limited insurance coverage.^7^ Unfortunately, the U.S. healthcare system benefits from the high prices of brand name PrEP prescriptions and clinical service fees that increase profit margins.

However, one way to increase PrEP access, initiation, and adherence among these key populations could be to establish a national program that eliminates barriers and ensures access to all. For example, a PrEP access initiative could be included within a national health care plan to all U.S. citizens if one were to exist. Many southern U.S. states have not adopted Medicaid expansion, which provides healthcare coverage for outpatient clinical services and laboratory fees to low-income patients. Therefore, many low-resourced individuals from key populations who could benefit from PrEP cannot access it in the U.S. South, where individual, social, clinical, and structural barriers are prevalent.

To fill some of the gaps for PrEP access in the U.S., the Department of Health and Human Services created the “Ready, Set, PrEP” program that provides free PrEP for qualified individuals along with education, clinicians, and resources such as diverse patient video testimonials about the PrEP program.^8^ The program also offers a navigation system that helps readers follow the steps to accessing PrEP, regardless of insurance coverage. Despite the helpful programming and assistance resources of the Ready Set PrEP program, gaps in accessing and maintaining care remain, particularly due to challenges that patients have covering follow-up clinical visits and lab costs, and travel costs for individuals in low resource contexts (e.g., the Southeastern U.S.). The result is a system in which many individuals can access and initiate PrEP use but cannot sustain consistent engagement. Since gaps in access, initiation, and adherence remain, a national PrEP program would provide more attention and details to address these deficiencies.

The time for a national PrEP access program that levels the playing field for the uninsured and those on Medicaid is now. The prevention science is irrefutable, consistent with other accepted prevention protocols such as mammography and colon cancer screening. Our communities are calling for improved access to PrEP as a sexual health service, and they deserve better than what is currently available to them.

To increase PrEP, the United States needs a program that supports access for low-income individuals, people who are on Medicaid, and people who lack insurance coverage. A new approach could overcome the challenges facing existing programs with similar goals, which is the animating concept behind the proposal from Killelea and colleagues in this issue of *JLME*.^9^ What are key considerations for effective implementation of a national PrEP program? On a policy level, the fact that PrEP has an “A” rating as a health prevention tool from the United States Preventive Services Task Force (USPSTF) has already increased access to insured individuals. This means that most private insurers and Medicaid expansion programs must cover related expenses such as labs and follow up visits without cost sharing. Extending this “A” rating to other forms of PrEP (i.e., long-acting injectables) will be important moving forward.

Additionally, creating a successful national PrEP program involves adaptability with options that are tailored to individual patients’ lives.^10^ Sexual risk is contextual and fluid. Therefore, our approaches must have the capacity, nuance, and flexibility to adjust to people’s needs. For increased access points, individuals should be able to obtain medication from several clinical service types: traditional brick and mortar clinics, telemedicine encounters, and even mobile services where clinician, pharmacist, or nurse-led visits can come to them. Implementing this program would involve coordination between private and public sectors, including involving community-based organizations and initiatives on the ground and in virtual/social media spaces so that communities will have widespread and equitable access. This will ensure that patients can utilize PrEP services in person, via mail order services, and at their local community clinics and spaces without barriers such as stigma and cumbersome prior-authorization forms serving as obstacles.

A national PrEP program must also work with the diverse mosaic of ways people can present when seeking services, such as creating an online “card” or QR code that can be easily accessed on a mobile device or online. As the proposed program focuses on individuals who are uninsured or on Medicaid, clinicians must be able to bill the appropriate program and fill in any gaps in coverage that are missing to make sure patients can initiate and continue PrEP with no additional out-of-pocket costs.^11^ Learning from the ongoing COVID-19 pandemic, related vaccine trials, and subsequent dissemination of prevention tools, we know that collaborations between the public and private sectors have the potential to be effective when it comes to public health interventions. HIV prevention is no different.

Similarly, a national initiative could and should offer standard 90-day prescriptions for persons starting PrEP, so that when life happens and someone misses a follow up appointment, medication delivery is not interrupted. For lab and clinical follow-ups, this national initiative should be linked and continually updated according to CDC and other national PrEP guidelines. Patients should have options that utilize tele-health follow-ups and remote ordering of labs for either at-home testing or in-person visit with a contracted vendor (LabCorp, Quest, etc.), and the frequency as dictated by scientifically determined standards. This would include recommended STI testing, urine pregnancy testing, and checking kidney function as indicated. The FDA has already approved two oral PrEP regimens for once daily use, as well as a long-acting injectable every two months regimen for HIV prevention, with alternative delivery models and methods in the works (e.g., long-acting subcutaneous, microbicides, implants, transdermal).^12^ Some clinicians prescribe “on-demand” dosing for their patients taking FTC/TDF, but this must be communicated as an “off label” use of PrEP in the United States at this time.^13^ Finally, a national program should be able to respond to patient needs. Currently there are brand name and generic versions of TDF/FTC available, and only one brand version of both TAF/FTC and long-acting cabotegravir. A national PrEP plan must be equipped to allow the proper prescribing of which option is best for the patient based on evidence and clinical considerations between individuals and their medical providers.

Given the outlined infrastructure and logistical concerns, it would be easy to forget about the personnel charged with the education, evaluation, prescription, and delivery of PrEP services. Research has demonstrated that individual level bias among healthcare staff can have an impact on choices to educate and prescribe PrEP to the populations who may benefit the most.^14^ For any national PrEP access program to be successful, diverse leadership representing the communities devastated by HIV and cultural humility training must be integrated into the fabric of the program.

Additionally, frequent evaluation, continuing education opportunities and a robust feedback service for persons accessing the program need to be incorporated to ensure that clinician bias does not serve as a barrier to equitable access and utilization of PrEP services.

The time for a national PrEP access program that levels the playing field for the uninsured and those on Medicaid is now. The prevention science is irrefutable, consistent with other accepted prevention protocols such as mammography and colon cancer screening. Our communities are calling for improved access to PrEP as a sexual health service,^15^ and they deserve better than what is currently available to them. While we may not have an overall national health care plan just yet, we have an opportunity to create a service that will ensure access to PrEP by utilizing the combined strengths of the public and private sectors. We can end the HIV epidemic, but only if we create a fair and equitable system for all.

## Notes

The authors do not have any conflicts of interest to disclose.
